# Xylan-Polyvinyl Alcohol Biopolymer Films Incorporated with *Zanthoxylum rhoifolium* Lam. Extract: Development, Characterization, and Antimicrobial Activity for Active Food Packaging

**DOI:** 10.3390/polym18091143

**Published:** 2026-05-06

**Authors:** Janine Siqueira Nunes, Brunna Emanuelly Guedes de Oliveira, Fernanda Matias Cariri Marques, Abrahão Alves de Oliveira, Elquio Eleamen Oliveira

**Affiliations:** 1 Department of Biotechnology, Federal University of Paraíba, João Pessoa 58051-900, PB, Brazil; janinesnunes@hotmail.com (J.S.N.); brunnaguedes151@gmail.com (B.E.G.d.O.); 2 Academic Unit of Biological Sciences, Health Center and Rural Technology, Federal University of Campina Grande, Patos 58708-110, PB, Brazil; fernandacariri20@gmail.com (F.M.C.M.); abrahao.alves@professor.ufcg.edu.br (A.A.d.O.F.); 3 Department of Biology, State University of Paraíba, João Pessoa 58071-470, PB, Brazil

**Keywords:** antimicrobial, extract, food packaging, polyvinyl alcohol, xylan, *Z. rhoifolium* Lam.

## Abstract

This study aimed to develop and characterize biodegradable films based on xylan and polyvinyl alcohol (PVA), incorporated with ethanolic extract of *Zanthoxylum rhoifolium* Lam., for application in active food packaging. A Box–Behnken experimental design was employed to optimize the concentrations of xylan, PVA, and glycerol, evaluating the mechanical properties of the films. The results indicated that glycerol reduced tensile strength and increased elongation at break, while higher concentrations of xylan and PVA promoted an increase in mechanical strength. FTIR and XRD analyses reveal structural change without the formation of new chemical structures. The *Z. rhoifolium* extract exhibited antibacterial activity with a minimum inhibitory concentration of 31.5 µg/mL for *Klebsiella pneumoniae*, increasing to 1000 µg/mL for *Enterococcus faecium*, *Enterobacter cloacae*, and *Escherichia coli*. In addition, hemolytic activity exceeded 80% at concentrations ≥ 1000 µg/mL, whereas concentrations up to 500 µg/mL can be considered safe, as hemolysis remained below 40%. The films incorporated with the extract showed antimicrobial activity, with emphasis on the formulation containing 60 mg of extract, which exhibited inhibition zones of up to 16 mm against *Escherichia coli* and 12 mm against *Enterococcus faecalis*. Thus, the results highlight the potential of functionalized xylan/PVA films as sustainable materials for the development of active packaging.

## 1. Introduction

The use of packaging within the food industry is fundamental to the efficient distribution and preservation of food products [1,2]. Conventional packaging is predominantly made from petroleum-based polymers, nonrenewable materials with low degradation rates and limited recyclability. Moreover, these synthetic polymers may affect food quality and pose health risks to consumers, as compounds from the packaging materials can migrate into food products [2,3]. In contrast, active biodegradable films have gained significant attention, both for their biodegradability and their ability to deliver bioactive compounds [4,5,6].

Given the environmental and health concerns associated with synthetic packaging, the development of biodegradable alternatives using naturally derived polymers has emerged as a promising strategy [7]. Xylan, the primary hemicellulose and one of the most abundant biopolymers in agricultural by-products, is a renewable resource with several properties that make it a promising candidate for applications in the pharmaceutical, food, and biomedical industries [8,9,10]. Its inherent biodegradability, biocompatibility, and film-forming capacity position xylan as an attractive base material for sustainable packaging. However, films made solely from isolated xylan often exhibit reduced mechanical strength, limiting their applications [11,12,13].

To overcome these limitations, polymer blends combining xylan with other polymers, like polyvinyl alcohol (PVA), are developed. PVA is widely used in food packaging due to its low permeability, biodegradability, and excellent film-forming capabilities [14,15]. Although studies by Wang et al. [12], Gao et al. [11], and Liu et al. [13] have demonstrated improvements in the mechanical properties of xylan–PVA films, none of these studies investigate in detail the influence of the relative proportions of xylan and PVA on film formation, nor do they address their antibacterial properties, which are crucial for active packaging applications.

Currently, research and development in food packaging is focused on active packaging. Studies show that the addition of essential oils and plant extracts can significantly enhance the antimicrobial, antioxidant, and physicochemical properties of composite materials [4,5,16,17]. Among various plant-based compounds with potential for incorporation into active packaging, *Zanthoxylum rhoifolium* Lam. stands out, known in Brazil as mamica-de-cadela, laranjeira-brava, and limãozinho. This tree is found in nearly all states, and its extracts are widely used for their pharmacological properties, including antibacterial, antiparasitic, antifungal, antipyretic, antiviral, gastrointestinal, and antimalarial activities [18,19,20,21]. Despite these promising properties, the incorporation of *Z. rhoifolium* extract into biopolymer films has not been previously explored.

Despite the progress in xylan–PVA blends, a significant gap remains in understanding how the proportions of these polymers, combined with the concentration of plasticizers influence the mechanical performance of the films. Furthermore, the potential of *Z. rhoifolium* extract to confer antimicrobial properties to these specific biopolymers matrices has not yet been established. Addressing these gaps is crucial for the design of optimized active packaging that meets both mechanical requirements and antimicrobial performance.

The hypothesis of this study is that the optimization of the xylan–PVA ratio, combined with the incorporation of *Zanthoxylum rhoifolium* extract, will result in a biopolymer film with superior mechanical flexibility and potent antimicrobial activity against foodborne pathogens. Based on these aspects, the objective of this study was to evaluate the influence of polymers and plasticizers on the formation of polymeric films through an experimental design, with the aim of incorporating *Zanthoxylum rhoifolium* Lam. extract for active packaging applications. The polymeric films were characterized through the analysis of mechanical properties, Fourier-transform infrared spectroscopy (FTIR), X-ray diffraction (XRD), and scanning electron microscopy (SEM). Additionally, the effect of the extract at different concentrations on the mechanical properties, hemolytic evaluation, and antibacterial properties of the films was also investigated.

## 2. Materials and Methods

### 2.1. Materials

Polyvinyl alcohol (molecular weight of 89,000–98,000 g/mol and degree of hydrolysis of 99%), acetic acid, methanol, and isopropanol were purchased from Sigma Aldrich (St. Louis, MO, USA); sodium hydroxide was purchased from Vetec Chemical (Duque de Caxias, Brazil); glycerol was purchased from ACS Científica (Sumaré, Brazil). Xylan was obtained after extraction from corn cobs as previously described by our group [22,23] with a yield of 16.53%.

### 2.2. Extract Preparation of Zanthoxylum rhoifolium *Lam.*

The plant material, consisting of the leaves of *Zanthoxylum rhoifolium* Lam., was individually dehydrated in a convection oven with circulating air at an average temperature of 40 °C for 72 h. The leaves were collected and identified by Prof. Dr. Maria de Fátima de Araújo Lucena. A voucher specimen (number 7552) was deposited at the Herbarium CSTR (Center for Health and Rural Technology) at the Federal University of Campina Grande, Patos Campus—PB. The dried leaves were then ground using a mechanical mill to obtain a powder. Ethanolic extraction was performed by subjecting the powder to exhaustive maceration for 72 h, with a proportion of 250 g of leaf powder for every 1 L of ethanol. The resulting extractive solutions were concentrated by evaporation under reduced pressure using a rotary evaporator, yielding the respective crude ethanolic extracts [24]. At the end of the process, phytochemical prospecting was performed (See Table S1 in the Supplementary Materials).

### 2.3. Experimental Design

The experimental design employed was a Box–Behnken design consisting of 17 runs, including 5 replicates at the central point, with three factors at three levels. The independent variables investigated were the concentrations of xylan, polyvinyl alcohol (PVA), and glycerol (Table 1). The dependent variables were the mechanical properties, specifically tensile strength (TS) and elongation at break (EB).

### 2.4. Preparation of Polymeric Films

**Scheme 1:**
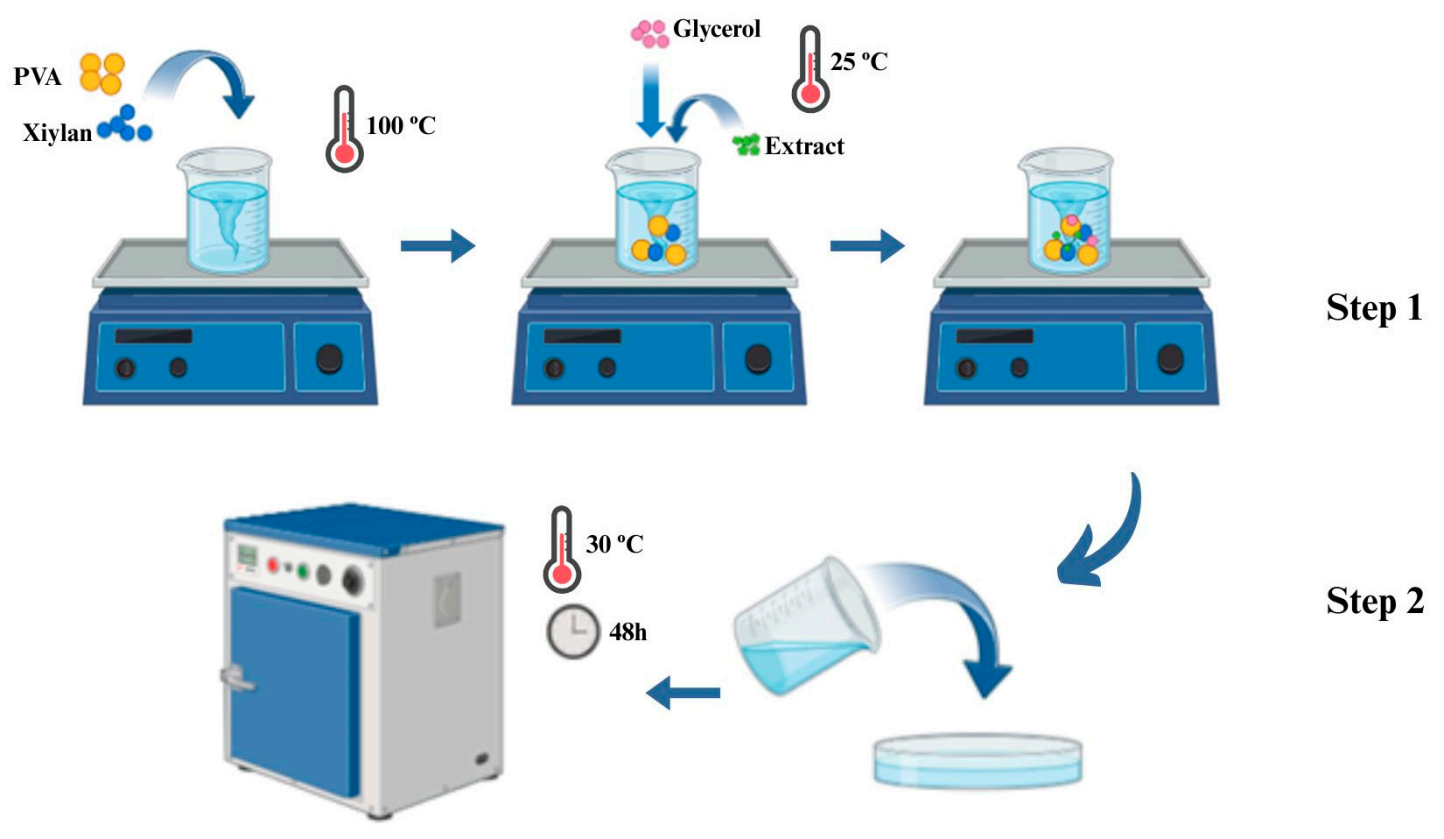
The preparation process of the films. Step 1: Preparation of the film-forming solution. Step 2: Casting and drying.

The polymeric films were prepared by the aqueous casting method, using different concentrations of xylan and PVA (100, 200, and 300 mg), as well as glycerol (50, 80, and 110 mg). Polymer dispersion was carried out at approximately 100 °C. After complete homogenization of the system, heating was turned off, and at room temperature, glycerol was added under stirring. The homogenized dispersions, with a volume of 15 mL, were cast into 40 mm diameter Teflon-coated plates and dried in an oven at 30 °C for 48 h. An experimental design approach was applied to optimize the concentrations of the polymers and plasticizers, using mechanical properties as response variables. After identifying the formulation with the best performance, the extract of *Z. rhoifolium* Lam. was incorporated at concentrations of 20 mg (E0120), 40 mg (E0140), and 60 mg (E0160), during the same stage of glycerol addition, in order to evaluate its influence on the film’s properties. The preparation process of the films was proposed in Scheme 1.

### 2.5. Thickness

The thickness of each film specimen was assessed using a digital micrometer, with values reported as the average of three measurements obtained from randomly selected points.

### 2.6. Mechanical Properties

The mechanical performance of the films in different formulations was evaluated through tensile strength tests, following the methodology described by Zhang et al. [25] and Gómez-Bachar et al. [26], with some modifications. In general, specimens measuring 30 × 15 mm were used, with three replicates tested for each formulation. The tests were performed on a Shimadzu AG-X universal testing machine (10 kN) at a crosshead speed of 5 mm/min and a gauge length of 10 mm.

### 2.7. Fourier Transform Infrared Spectroscopy (FT-IR)

FT-IR analysis was performed using a Shimadzu IRSpirit spectrometer equipped with an Attenuated Total Reflectance (ATR) module. Spectra were recorded in the range of 4000–400 cm^−1^ with a resolution of 8 cm^−1^.

### 2.8. X-Ray Diffraction (XRD)

X-ray diffraction patterns of the films were obtained using a Shimadzu XRD-6100 diffractometer with a vertical goniometer. The measurements were carried out over a 2θ range of 5–60° at a scanning rate of 2°/min. Prior to analysis, the samples were dried and stored in a desiccator.

### 2.9. Scanning Electron Microscopy (SEM)

The surface morphology of the films was analyzed using a Tescan Vega III scanning electron microscope (SEM). Prior to analysis, the film samples were dried overnight in an oven and then mounted on metal stubs using double-sided adhesive tape. Subsequently, all film specimens were sputter coated with a thin layer of gold to enhance conductivity and prevent surface charging of electrons.

### 2.10. Determination of Minimum Inhibitory Concentration (MIC) and Minimum Bactericidal Concentration (MBC)

The MIC was determined using the microdilution method in 96-well plates containing Mueller–Hinton broth and the extract at concentrations ranging from 1024 to 16 μg/mL. An aliquot of 10 μL of the inoculum was added to each well, and positive and negative controls were included. After 24 h of incubation at 37 °C, the initial reading was performed. Then, 0.01% resazurin was added for colorimetric evaluation. The MIC was defined as the lowest concentration that inhibited bacterial growth, as indicated by the maintenance of the blue color of the indicator [27,28,29]. After determining the MIC, 10 µL of three subsequent dilutions of the MIC were inoculated into Mueller–Hinton broth and incubated at 37 °C for 24 h. Then, 20 μL of resazurin was added, followed by another 24 h incubation. The MBC was defined as the lowest concentration that completely inhibited bacterial growth, confirmed by the absence of color change in the indicator [30,31].

### 2.11. Determination of Minimum Inhibitory Concentration for Adhesion (MICA)

MICA was determined according to the literature [32], with modifications. The bacterial strain was cultured in Mueller–Hinton broth at 37 °C. A volume of 0.9 mL of the subculture was transferred to test tubes containing 0.1 mL of the compound at different concentrations (up to 1:1024) and 5% sucrose. The tubes were incubated at 37 °C for 24 h at a 30° angle. The result was interpreted based on the absence of visible bacterial adhesion to the inner walls of the tubes after gentle shaking. A 0.12% chlorhexidine solution was used as a positive control. MICA was defined as the lowest concentration that inhibited bacterial adhesion.

### 2.12. Evaluation of Cytotoxicity in Human Erythrocytes

Human erythrocytes (types A, B, and O) were obtained from healthy donors, following biosafety guidelines. Blood samples were diluted in 0.9% NaCl (1:30) and centrifuged at 2500 rpm for 5 min. This process was repeated twice to isolate the erythrocytes. The final pellet was resuspended in 0.9% NaCl to obtain a 0.5% suspension. Extract samples at concentrations ranging from 50 to 1000 μg/mL were added to 2 mL of the erythrocyte suspension, for a final volume of 2.5 mL. The following controls were used: erythrocytes in 0.9% NaCl (negative control, 0% hemolysis) and erythrocytes in 1% Triton X-100 (positive control, 100% hemolysis). Samples were incubated at 22 ± 2 °C for 1 h with gentle shaking, then centrifuged (2500 rpm, 5 min). Hemolysis was quantified by spectrophotometry at 540 nm [33].

### 2.13. Antibacterial Activity of Films Incorporated with Zanthoxylum rhoifolium *Lam.* Extract

The antibacterial activity of the films was evaluated using the well diffusion method on Mueller–Hinton agar. Wells, 6 mm in diameter, were made in a solid medium and filled with film disks at different extract concentrations. The plates were inoculated with standardized bacterial suspensions (1 × 10^6^ CFU/mL) and incubated at 35 °C for 24 h. Halos ≥ 6 mm were considered indicative of antibacterial activity [29,34].

### 2.14. Ethics Statement

All procedures were conducted in accordance with relevant national and institutional guidelines. The study protocol was reviewed and approved by the Research Ethics Committee of the University Center of Patos (UNIFIP), Brazil, under Protocol Number 6.076.256; 24 May 2023.

### 2.15. Statistical Analysis

Mechanical strength tests were performed in triplicate, and the results were expressed as mean ± standard deviation. The data were statistically analyzed using analysis of variance (ANOVA) to assess the significant effects of different independent variables on the response factors, performed using Design-Expert^®^ software (version 13, Stat-Ease Inc., Minneapolis, MN, USA). Post hoc multiple comparisons were conducted using Tukey’s test in Jamovi software (version 2.6.26), and a *p*-value of <0.05 was considered statistically significant, with a 95% confidence interval.

## 3. Results

According to the experimental design, 17 test formulations were prepared and characterized in terms of thickness and mechanical properties. All characterization experiments were conducted in triplicate, and the results are presented in Table 2.

**Figure 1:**
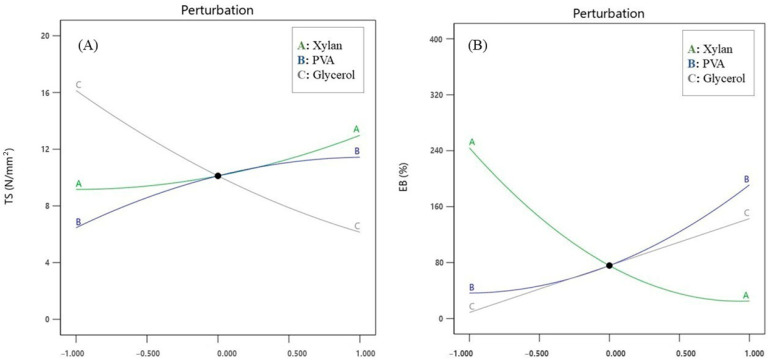
Effect of xylan, PVA, and glycerol concentrations on the TS (**A**) and EB (**B**) of the films.

The influence of each factor (xylan, PVA, and glycerol) on the mechanical properties was evaluated using a Box–Behnken design. Figure 1 illustrates the effects of the independent variables on TS and EB, respectively.

The TS values ranged from 2.82 to 22.30 MPa. Significant factors included xylan (A), PVA (B), glycerol (C), and the interaction between xylan and glycerol (AC). The quadratic model for TS was found to be highly significant (F-value = 31.08; *p* < 0.0001), with a coefficient of determination R^2^ of 0.9756. This indicates that the model accounts for 97.56% of the variability in TS, demonstrating an excellent fit to the experimental data. The adjusted R^2^ was 0.9442, and the adequate precision was 24.3992, well above the desirable threshold of 4, confirming an adequate signal-to-noise ratio as determined using Design-Expert^®^ 13 software (see Tables S2 and S3 and Equation (S1)).

As shown in Figure 1, glycerol concentration had a strong negative effect on TS, leading to a significant reduction as its concentration increased. This behavior is characteristic of plasticizers, such as glycerol, which reduces intermolecular interactions between polymer chains, thereby increasing flexibility but decreasing the mechanical strength of the films [3,35]. This effect is exemplified in formulation 11, which exhibited a relatively low TS (8.72 ± 0.61 MPa), while the EB was high (365.04 ± 95.75%). Similarly, formulation 5, which had a low content of structural polymers, exhibited one of the lowest TS values (4.26 ± 1.21 MPa).

On the other hand, increasing the concentrations of PVA and xylan resulted in higher TS, as seen in formulations 10 and 15, which showed TS values of 18.46 ± 6.66 MPa and 15.59 ± 1.79 MPa, respectively. This effect can be attributed to PVA’s ability to form denser and more cohesive polymer networks, thereby enhancing the structural integrity of the material [36], in combination with xylan, which, as a structural polysaccharide, contributes to the rigidity of the polymer matrix.

EB values showed high variability, peaking at 438.2%. The primary influences were xylan (A), the interaction between xylan and PVA (AB), and the quadratic term (A2) (see Tables S2 and S4 and Equation (S2)).

The quadratic model for elongation at break (EB) was also significant (F-value = 7.35; *p* = 0.0077), with an R^2^ of 0.9043. However, the model exhibited a significant “Lack of Fit” (*p* < 0.0001) and a negative predicted R^2^ (−0.5267), suggesting that while the model identifies trends, it may not be suitable for precise predictions across the entire design space. Diagnostic tools, including Cook’s Distance and DFFITS plots, identified Run 4 as a severe outlier, exerting disproportionate influence on the model parameters.

After excluding Run 4, the quadratic model was re-evaluated. This refinement led to a remarkable improvement in the model’s reliability, with the R^2^ increasing to 0.9942 and the adjusted R^2^ to 0.9875. Most importantly, the predicted R^2^ shifted from a negative value to 0.9020, demonstrating a good degree of predictive power for new observations. Although the lack of fit remained significant (*p* = 0.0042), the high Adequate Precision (41.0317) and the strong correlation between experimental and predicted values justify the use of this model for navigating the design space and optimizing the film formulation. Such residual lack of fit is often attributed to the inherent structural complexity and non-linear interactions within hemicellulose-based polymeric matrices.

According to Figure 1B EB responded inversely to TS. An increase in xylan concentration reduced EB, likely due to the increased structural rigidity caused by the dense hydrogen bonding networks that restrict polymer chain mobility [14]. This is exemplified by formulation 10, which, despite having the highest TS, showed an EB of only 30.92 ± 2.17%, indicating a stiffer and less elastic material. Interestingly, the interaction between xylan and PVA (AB) was also significant and negative (−69.17), suggesting that the blend becomes less flexible as both polymer concentrations increase, likely due to increased molecular crowding and restricted chain mobility (See Figures S1–S6). In contrast, higher glycerol concentrations resulted in increased flexibility, as observed in formulations 11 (365.04 ± 95.75%) and 14 (438.20 ± 257.04%), typical of plasticized systems. This finding is consistent with the literature [37], which reported that the addition of glycerol increased EB in arrowroot starch films.

### 3.1. Variable Optimization

A numerical optimization based on desirability function was performed to predict the optimal levels of these variables to achieve the best outcomes for all responses, thereby maximizing both TS and EB. The optimal condition was achieved with 300 mg of PVA, 100 mg of xylan, and 70 mg of glycerol, with an overall desirability of 0.829, which corroborates the findings of Gao et al. [11], who reported that PVA/xylan blends exhibited improved TS and EB values at a 3:1 ratio.

Optimization of these parameters is crucial, as materials intended for packaging applications must meet multiple performance criteria simultaneously. For example, the material should demonstrate high TS to ensure structural integrity, as well as high elongation to provide flexibility [6]. By carefully selecting the appropriate concentrations of PVA, xylan, and glycerol, it is possible to develop packaging films that fulfil these requirements. Accordingly, new films (SE01) were developed under the optimal conditions, incorporating the extract at varying concentrations and subjected to mechanical strength analysis and thickness measurements, as shown in Table 3. The model predicted values of 358% for EB and 12 MPa for TS, indicating that this composition should theoretically maximize flexibility while maintaining adequate mechanical resistance.

However, the experimental validation revealed substantial deviations from the predicted responses. The experimental TS reached 19.57 MPa, exceeding the model prediction. This divergence suggests that the quadratic model was able to capture the strengthening effect of the formulation but was less accurate in predicting the extensibility.

The measured EB was nearly 47% lower than the predicted value, it remained within a functional range for flexible polymeric films. This variation is frequently reported in films, since this property is highly sensitive to factors such as relative humidity, due to the hydrophilic nature of the polymer, as well as to the film microstructure, including plasticizer distribution, structural heterogeneity, and the presence of defects. Small changes in these parameters may result in significant variations in mechanical behavior [35,38]. However, the model successfully identified the region of maximum performance, as the optimized film demonstrated superior mechanical balance compared to most of the preliminary runs.

Despite these limitations, the validation confirms that the optimized formulation indeed provides a balanced mechanical profile, combining high extensibility with above-average tensile strength, even if the absolute EB values were overestimated by the model. In addition, the mechanical properties observed in the optimized film are comparable to other xylan-based composite films report in the literature that present a TS of 10–22 MPa and EB of 45–258% [9,11,39] and superior to xylan films isolated [40].

The thickness and mechanical properties of xylan/PVA films are presented in Table 3. The thickness of the neat xylan/PVA films was 0.07 mm, which increased significantly after the incorporation of different concentrations of the extract, reaching 0.11 mm (20 mg), 0.14 mm (40 mg), and 0.13 mm (60 mg). These results are consistent with the findings of Shankar, Khodaei, and Lacroix [41], who reported that incorporating essential oils into films led to an increase in thickness. This increase in thickness is consistent with the incorporation of extract solids into the polymer matrix, which adds mass and volume to the film structure. The thicker films reflect the physical presence of polyphenolic compounds and other extract constituents distributed throughout the xylan–PVA network, further supporting the interpretation that the extract functions as a filler material. In addition to the solid content, the interaction between the polymer and the extract directly influences the thickness of the polymer films. This increase in thickness has also been observed in composite materials [42,43]. As the concentration of polyphenols increases, the interactions between the extract and the polymers intensify, resulting in a stronger bond. In this context, polyphenols can act as bridges, establishing bonds with multiple molecules. Thus, these additives have the potential to form compact structures, contributing to the increase in film thickness through multiple interactions.

The incorporation of the extract into xylan/PVA films produced a statistically significant effect on TS values (*p* < 0.05), whereas EB values remained unaffected (*p* > 0.05). The highest TS value was observed in SE01 (film without extract), with a value of 19.57 MPa. As the extract concentration increased, a gradual decrease in TS was observed. Films containing 40 and 60 mg of *Z. rhoifolium* extract showed a significant reduction in TS. The polyphenolic compounds present in *Z. rhoifolium* extract—including alkaloids, flavonoids, and tannins—contain multiple hydroxyl and carbonyl functional groups capable of competing for hydrogen-bonding sites on the polymer chains. This effect has been attributed to the disruption of intermolecular interactions, such as hydrogen bonds, which reduce mechanical strength without necessarily increasing the flexibility of the polymer chain [44]. These findings are consistent with previous studies, which have reported that the incorporation of plant extract into polymeric films generally interferes with the polymer network cohesion [45,46,47].

In the present study, the absence of a statistically significant change in EB despite the marked reduction in TS suggests that the extract does not substantially alter the segmental mobility or flexibility of the polymer chains. Instead, the extract particles act as rigid or semi-rigid inclusions that disrupt load-bearing hydrogen bonds without conferring the enhanced chain mobility characteristic of plasticization. Similar behavior has been reported in polymeric films containing essential oils; TS decreases sharply with increasing essential oil concentration, while EB values do not show significant variations [44].

### 3.2. FTIR

**Figure 2:**
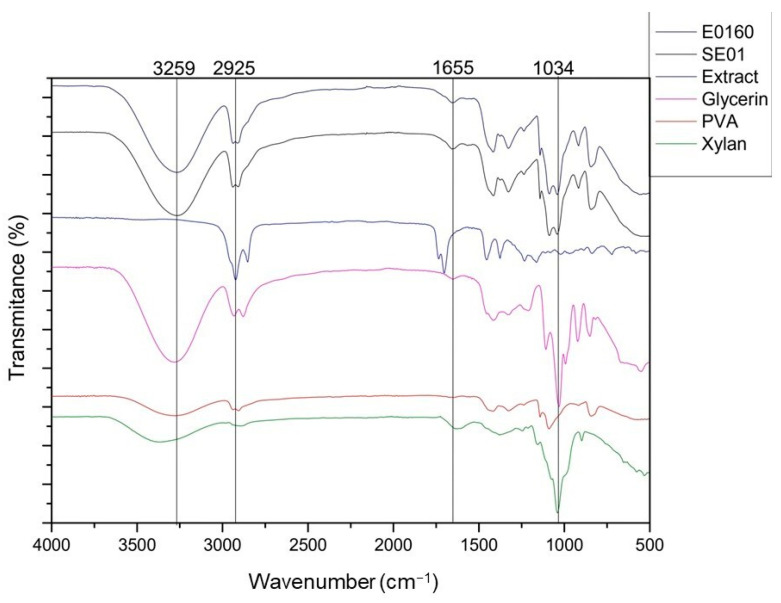
FTIR spectra of xylan, PVA, glycerol, the *Z. rhoifolium* Lam. extract, and films with and without the *Z. rhoifolium* Lam. extract.

FTIR-ATR analyses were performed to investigate the interactions between the formulation components. The analyses were conducted on both the raw materials and the films, as shown in Figure 2. The structure of the extracted xylan was confirmed by comparing the obtained spectra with previously published data from our research group, which showed absorption bands at 3375, 2905, 1628, 1389, 1034, and 898 cm^−1^, consistent with those described in the literature [23]. The spectra of polyvinyl alcohol (PVA) and glycerol also exhibited typical bands, in agreement with the literature data [48,49]. The ethanolic extract displayed multiple bands, particularly in the regions between 2929 and 1450 cm^−1^, attributed to CH stretching vibrations from CH_2_ and CH_3_ groups [50]. Additionally, bands at 1692 and 1456 cm^−1^ were assigned to aromatic C=C vibrations and the presence of amide groups [50,51]. The FT-IR spectra of xylan/PVA films exhibited characteristic absorption peaks at 3259 cm^−1^ (attributed to O–H stretching vibrations) 2925 cm^−1^ (C–H stretching of alkanes), 1655 cm^−1^ (residual acetyl groups in xylan), and 1034 cm^−1^ and 1042 cm^−1^ (C–O stretching) [9,12,13]. Specifically, the shift in the O–H stretching peak from 3375 cm^−1^ in pure xylan to approximately 3259 cm^−1^ in the composite films provides direct evidence of hydrogen bonding among xylan, PVA [12]. The spectra obtained for the SE01 and E0160 films exhibited similar profiles, suggesting that the addition of the extract did not induce the formation of new chemical bonds or significant shifts in characteristic absorption bands compared to the control film, indicating that the extract did not chemically crosslink the polymer chains or form covalent linkages within the matrix [52,53].

### 3.3. X-Ray Diffraction

**Figure 3:**
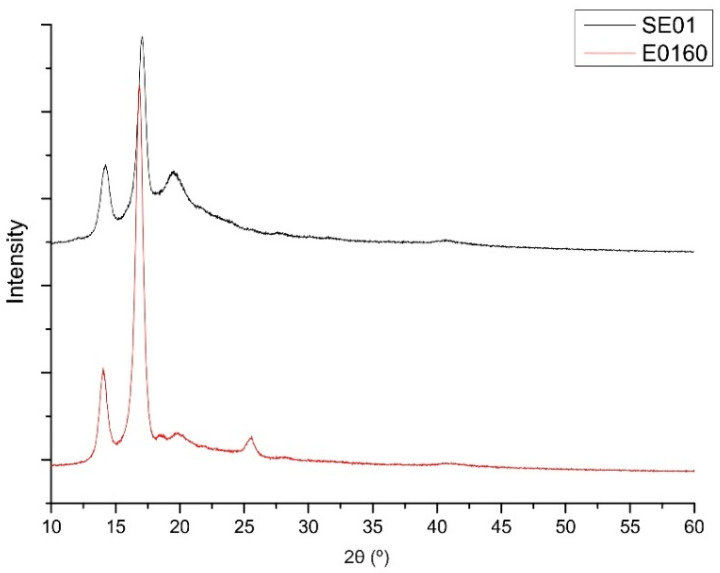
X-ray diffraction of blending films.

The crystalline structure and molecular organization of the films, both with and without *Zanthoxylum rhoifolium* extract, were investigated using X-ray diffraction, as shown in Figure 3. These diffractograms were compared with the peaks of PVA and xylan used in this study and previously published [54,55,56,57]. The diffractograms revealed three main diffraction peaks at 2θ = 14.10°, 17.01°, and 19.65°, indicating the semi-crystalline nature of the films, associated with the semi-crystalline nature of PVA, consistent with the results of Liu et al. [13] and Gao et al. [11]. The presence of xylan, typically an amorphous or poorly crystalline polysaccharide, is evidenced by the broad amorphous halo underlying the sharper PVA reflections, suggesting that xylan is well-dispersed within the PVA matrix, forming a predominantly semi-crystalline network governed by the synthetic polymer.

Upon the incorporation of extract, a new diffraction peak appears at 2θ ≈ 25.48°. This signal suggests the presence of crystalline bioactive compounds from the extract, such as specific alkaloids or flavonoids, which may either be suspended in the polymeric matrix or form crystalline micro-domains during the solvent casting process. Furthermore, an increase in peak intensity at 17.01° was observed following extract addition, suggesting that the extract components act as nucleating agents or promote a more efficient packing of the polymer’s chains through specific physical interactions.

The changes in crystallinity observed in the diffractogram without significant alterations in the chemical structure were observed in the FTIR spectra, indicating that the extract primarily induced physical reorganization within the polymer. This dual analysis confirms that the *Zanthoxylum rhoifolium* extract is physically immobilized within the xylan–PVA blend, maintaining its chemical integrity while modifying the film’s crystalline morphology.

### 3.4. SEM

**Figure 4:**
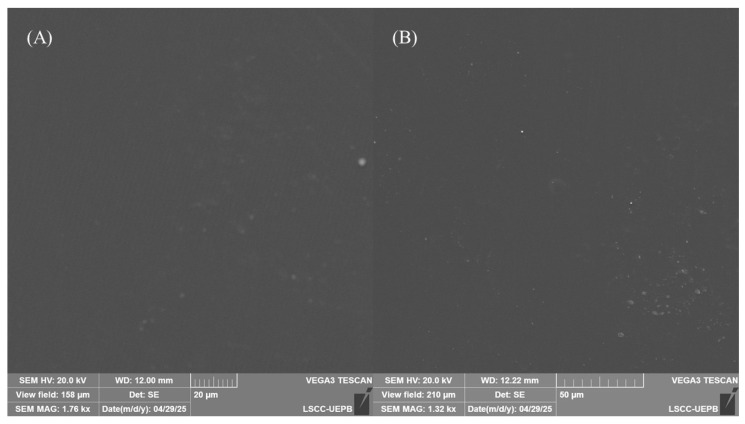
SEM micrographs of the surface of xylan/PVA films. (**A**) Without extract; (**B**) with the highest extract concentration.

Figure 4 shows the surface morphology images obtained by SEM of the film without extract (SE01) and the film with the highest extract concentration (E0160). The SE01 film exhibits a smooth and continuous surface with no visible pores, indicating a high degree of compatibility between these polymers, with no separation between the polymeric phases, indicating excellent compatibility between PVA and xylan molecules. Liu et al. (2023, 2024), studying xylan/PVA systems, observed films with a similar smooth morphology and few or no visible pores [13,39]. In contrast, the film containing the plant extract (E0160) exhibited a little more heterogeneous surface with increased roughness. A similar trend has been widely reported for PVA-based films incorporated with plant extracts. For instance, Singh, Gaikwad, and Lee (2018), observed slight morphological changes in PVA films containing basil leaf extract [58], while Tanwar et al. (2021) reported increased surface roughness in PVA films incorporated with coconut shell extract [59]. These findings agree with the results of the present study, suggesting that the presence of bioactive compounds promotes localized aggregation and disrupts surface uniformity.

In the present study, the rougher morphology of E0160 likely results from partial aggregation of extract components and their interactions with the hydrophilic polymer chains. Despite this increase in surface irregularity, no cracks or large-scale phase separation were observed, indicating that the polymer matrix remained structurally continuous. This morphological behavior is consistent with the mechanical results, where extract incorporation reduced tensile strength but did not significantly affect elongation at break. Together with the XRD findings of increased crystallinity and the FTIR evidence of preserved chemical structure, the SEM results suggest that the extract induces physical rearrangements within the matrix rather than chemical modifications.

### 3.5. MIC and MBC

The following results were obtained for the MIC and MBC of the *Z. rhoifolium* extract against Gram-positive bacteria associated with food contamination: Enterococcus faecium exhibited an MIC of 1000 µg/mL. In contrast, Enterococcus faecalis and Staphylococcus saprophyticus showed MICs of 500 µg/mL (Table 4). According to Table 4, the antibacterial activity of *Z. rhoifolium* extract against Gram-negative bacteria was also observed. Enterobacter cloacae and *Escherichia coli* strains presented MICs of 1000 µg/mL. For *Klebsiella pneumoniae*, the MIC was 31.5 µg/mL. For all bacterial strains tested, it was not possible to experimentally determine the MBC, as the values were above 1000 µg/mL.

According to the classification proposed by Sartoratto et al. [60], antimicrobial activity can be classified as strong when the MIC is up to 500 µg/mL, moderate for MICs ranging from 600 to 1500 µg/mL, and weak for MICs above 1500 µg/mL. Based on this criterion, the results obtained in this study indicate that the extract of *Zanthoxylum rhoifolium* exhibited strong activity specifically against *K. pneumoniae*, while showing moderate activity against the other tested strains. Several experimental studies show that certain natural extracts or antimicrobial compounds exhibit greater inhibition against *K. pneumoniae* than in other bacterial species. This can be explained by cellular permeability, as Klebsiella possesses a membrane that is less selective than other bacteria, such as Pseudomonas aeruginosa, facilitating the entry of compounds. Furthermore, in *Klebsiella pneumoniae*, an absence of such efficient efflux systems is observed, causing compounds to accumulate more easily in the cell; additionally, this species of bacteria possesses conserved metabolic targets for many agents (especially natural ones) to act upon, such as the cell wall and protein synthesis [61,62,63].

Furthermore, a compound is classified as bactericidal or bacteriostatic based on the MBC/MIC ratio: if the ratio ranges from 1:1 to 2:1, the compound is considered bactericidal; if the ratio exceeds 2:1, it is classified as bacteriostatic [64]. According to the MBC results, the *Z. rhoifolium* extract demonstrated bacteriostatic activity against all tested strains, including both Gram-positive and Gram-negative bacteria.

Previous studies have demonstrated the antibacterial potential of *Zanthoxylum rhoifolium*. For instance, it was reported that the crude leaf extract of *Z. rhoifolium* exhibits moderate antibacterial activity against both Gram-positive and Gram-negative bacteria, with particularly notable effects on Gram-negative strains [65]. This is significant, considering that many natural compounds typically show greater activity against Gram-positive bacteria. Another study evaluated the crude methanolic bark extract of *Z. rhoifolium* and reported MIC values of 250 µg/mL against *Staphylococcus epidermidis* and 500 µg/mL against *Klebsiella pneumoniae*. In the same work, the antimicrobial activity was attributed primarily to the presence of benzophenanthridine alkaloids [66]. Beyond *Z. rhoifolium*, the antimicrobial potential of other *Zanthoxylum* species within the Rutaceae family has also been investigated. The crude extract of *Zanthoxylum paracanthum* showed activity against *Staphylococcus aureus*, *Escherichia coli*, and *K. pneumoniae* [67], while *Zanthoxylum gilletii* demonstrated activity against *E. coli*, *K. pneumoniae*, and *Pseudomonas aeruginosa* [68]. These findings highlight the broader antimicrobial potential of the *Zanthoxylum* genus against clinically relevant pathogens.

### 3.6. Minimum Inhibitory Concentration for Adhesion (MICA)

Bacterial biofilm formation is one of the main challenges faced by the food industry. Conventional control strategies, such as the use of chemical preservatives and intensive processing, often reduce product appeal among consumers, who increasingly prefer minimally processed foods free from synthetic additives [69]. In this context, the search for natural compounds with antibiofilm potential has become a crucial strategy.

The determination of the MICA of *Zanthoxylum rhoifolium* extract demonstrated its ability to inhibit *Escherichia coli* adhesion to the glass tube wall at a dilution of up to 1:8 (Table 5). This result is particularly relevant for biofilm control, as it interferes with a critical stage of the process: the irreversible attachment of bacteria to surfaces [70]. Additional studies on other *Zanthoxylum* species further support their antibiofilm potential. For instance, bark and fruit extracts from *Zanthoxylum gilletii* were evaluated against *E. coli* strains and demonstrated inhibition of biofilm formation at the same 1:8 dilution [71]. The authors highlight that using plant extracts and natural compounds capable of disrupting biofilm development by pathogenic bacteria represents a promising strategy in combating microbial resistance, as biofilms function as protective barriers that enhance bacterial survival under adverse conditions.

### 3.7. Hemolytic Activity

The toxicity of bioactive compounds must be evaluated to ensure minimal adverse effects. One study used red blood cells as a model to assess drug toxicity, classifying hemolytic activity by percentage as follows: low (0–40%), moderate (40–80%), and high (above 80%) [33].

As shown in Table 6, the analysis of the hemolytic potential of *Zanthoxylum rhoifolium* extract revealed low hemolysis levels, ranging from 5.73% to 36.70% at concentrations between 50 and 500 μg/mL across all tested blood types. These values are consistent with the previously determined MIC_50_ against bacterial strains associated with food contamination. At the highest concentration tested (1000 µg/mL), a significant increase in hemolysis was observed, exceeding 80%, with variations depending on the blood type. However, these results are in line with previous studies, such as the work of Leitão et al. [72], who evaluated the hemolytic activity of *Z. rhoifolium* extract on erythrocytes from blood group O and found hemolysis levels below 10% at concentrations up to 400 μg/mL. This suggests that, at lower concentrations, the extract exhibits minimal hemolytic effects, supporting its potential as a safe and effective antimicrobial agent.

### 3.8. Antibacterial Activity of the Films

The qualitative assessment of the antimicrobial activity of films incorporated with *Z. rhoifolium* extract, as presented in Table 7, demonstrated that the formulation containing the highest extract concentration exhibited the strongest antibacterial effect, with inhibition zones measuring 16 mm against *E. coli* (Ec43) and 12 mm against *E. faecalis* (Ef48).

Possible causes for the results found with the Klebsiella pneumoniae strain are limited diffusion of hydrophobic active compounds through the hydrophilic matrix [73], and partitioning effects [74]. Studies reported in the literature involving polymeric films incorporated with plant extracts demonstrate the broad potential of these systems, although direct numerical comparisons of inhibition zones must be interpreted with caution due to differences in polymer matrices, microbial strains, and assay methodologies. For instance, PVA films containing *Uncaria gambir* extract were investigated, showing inhibition zones of 9.9 ± 0.9 mm against *E. coli* strains [16]. Similarly, PVA films functionalized with gold nanoparticles exhibited inhibition halos of up to 13 mm against *E. coli* [75]. Despite the inherent variations between these systems and the current study, the inhibition halos observed for *Z. rhoifolium* films suggest a competitive antimicrobial performance. In addition to PVA, other polymers have also been employed as active matrices in combination with plant extracts. Chitosan films containing *Allium tuberosum* extract were evaluated, observing inhibition zones ranging from 14.91 ± 0.29 mm to 18.79 ± 0.37 mm against strains of *E. coli*, *Bacillus cereus*, *Staphylococcus aureus*, and *Salmonella typhimurium* [17]. While these systems involve different chemical interactions and release kinetics, collectively they reinforce the viability of plant extracts as effective agents in biodegradable matrices for active packaging applications.

## 4. Conclusions

Biodegradable xylan/PVA films incorporated with *Z. rhoifolium* Lam. extract exhibited tunable mechanical properties and significant antimicrobial activity, establishing their potential as viable materials for application in active food packaging. Optimization of the formulations enabled the development of films with a balance between tensile strength and flexibility, with polymer and plasticizer concentrations being key determinants of mechanical performance. The incorporation of the plant extract did not compromise the structural integrity of the films, as confirmed by FTIR analysis and XRD. The extract exhibited bacteriostatic activity against foodborne pathogenic microorganisms, along with low hemolytic toxicity. Films containing 40 mg and 60 mg of extract displayed inhibition zones against *E. coli*. Future research should evaluate the application of these active films in food matrices susceptible to enteropathogenic contamination, such as fresh poultry or minimally processed vegetables. Specifically, storage studies under refrigeration should be conducted to monitor shelf-life indicators, including lipid oxidation levels and pH variations, ensuring the films maintain their antimicrobial efficacy in complex food environments.

## Figures and Tables

**Table 1:** Experimental design Box–Behnken.

Independent Variables	Levels		
	−1	0	1
Xylan	100	200	300
PVA	100	200	300
Glycerol	50	80	110

**Table 2:** Box–Behnken experimental design, tensile strength (TS), elongation at break (EB) and thickness of different films.

	PVA (mg)	Xylan (mg)	Glycerol (mg)	TS (MPa)	EB (%)	Thickness (mm)
1	100	300	80	8.84 ± 1.59	42.23 ± 7.57	0.22 ± 0.03
2	200	200	80	9.68 ± 0.87	75.55 ± 23.87	0.23 ± 0.02
3	200	200	80	9.45 ± 0.97	70.74 ± 10.82	0.18 ± 0.01
4	300	200	110	7.60 ± 1.95	54.59 ± 9.76	0.26 ± 0.03
5	100	100	80	4.26 ± 1.21	146.43 ± 74.17	0.09 ± 0.01
6	200	200	80	10.62 ± 1.22	80.67 ± 11.46	0.19 ± 0.02
7	100	200	50	13.89 ± 0.61	40.35 ± 12.51	0.12 ± 0.01
8	200	100	50	12.36 ± 0.91	102.8 ± 29.08	0.14 ± 0.01
9	200	200	80	9.85 ± 1.84	73.67 ± 13.52	0.18 ± 0.02
10	200	300	50	22.30 ± 0.41	30.92 ± 2.17	0.21 ± 0.02
11	200	100	110	8.37 ± 0.16	365.04 ± 95.75	0.17 ± 0.01
12	100	200	110	2.82 ± 1.20	34.38 ± 12.63	0.18 ± 0.01
13	200	300	110	5.37 ± 1.54	45.67 ± 10.38	0.24 ± 0.02
14	300	100	80	11.38 ± 1.55	438.2 ± 257.04	0.22 ± 0.02
15	300	200	50	15.59 ± 1.79	60.48 ± 8.51	0.26 ± 0.02
16	200	200	80	11.00 ± 1.52	72.28 ± 17.25	0.19 ± 0.01
17	300	300	80	15.13 ± 1.65	57.33 ± 10.43	0.27 ± 0.03

PVA = Polyvinyl alcohol; TS = tensile strength; EB = elongation at break.

**Table 3:** Mechanical properties of optimized films incorporated with *Z. rhoifolium* extract.

Sample	TS (MPa)	EB (%)	Thickness (mm)
SE01	19.57 ± 3.69 ^a^	191.42 ± 63.02 ^a^	0.07 ± 0.01 ^a^
E0120	15.04 ± 2.42 ^ab^	234.03 ± 38.57 ^a^	0.11 ± 0.02 ^b^
E0140	10.82 ± 1.56 ^b^	131.96 ± 27.05 ^a^	0.14 ± 0.01 ^b^
E0160	10.92 ± 2.06 ^b^	200.36 ± 26.90 ^a^	0.13 ± 0.02 ^b^

Legend: TS = tensile strength; EB = elongation at break SE01 (no extract) E0120 (20 mg extract) E0140 (40 mg extract) E0160 (60 mg extract). Different letters (a, b) in the same column mean significant differences (*p* < 0.05) between samples.

**Table 4:** MIC and MBC of *Z. rhoifolium* extract against Gram-positive and Gram-negative bacterial strains.

Strains	MIC (μg/mL)	MBC (μg/mL)
Ea44	1000	-
Ef48	500	-
Sa47	500	-
Ecl43	1000	-
Ec43	1000	-
Kp52	31.5	-

Legend: Ea44: Enterococcus faecium; Ef48: Enterococcus faecalis; Sa47: Staphylococcus saprophyticus; Ecl43: Enterobacter cloacae; Ec43: Escherichia coli; Kp52: Klebsiella pneumoniae. (-) was not determined experimentally.

**Table 5:** Minimum inhibitory concentration for adhesion (MICA) of *Z. rhoifolium* extract and 0.12% chlorhexidine digluconate against the *E. coli* strain.

Extract							
1:1	1:2	1:4	1:8	1:16	1:32	1:64	1:128
−	−	−	−	+	+	+	+
0.12% Chlorhexidine Digluconate							
1:01	1:02	1:04	1:08	1:16	1:32	1:64	1:128
−	−	−	−	−	−	−	−

**Table 6:** Percentage of hemolysis caused by the crude extract of *Z. rhoifolium* in human erythrocytes (ABO blood group system).

Blood Type	Concentration (μg/mL)	% Hemolysis
Blood A	50	7.19
Blood A	100	8.95
Blood A	500	35.34
Blood A	1000	93.06
Blood B	50	6.99
Blood B	100	8.14
Blood B	500	35.17
Blood B	1000	100
Blood O	50	5.73
Blood O	100	6.89
Blood O	500	36.70
Blood O	1000	100

**Table 7:** Inhibition zone diameters of films incorporated with *Z. rhoifolium* extract.

Bacterial Strains	SE01	E0120	E0140	E0160
Ea44	0 mm	0 mm	0 mm	0 mm
Ef48	0 mm	0 mm	0 mm	12 mm
Sa47	0 mm	0 mm	0 mm	0 mm
Ecl43	0 mm	0 mm	0 mm	0 mm
Ec43	0 mm	0 mm	14 mm	16 mm
Kp52	0 mm	0 mm	0 mm	0 mm

Legend: Ea44: *Enterococcus faecium*; Ef48: *Enterococcus faecalis*; Sa47: *Staphylococcus saprophyticus*; Ecl43: *Enterobacter cloacae*; Ec43: *Escherichia coli*; Kp52: *Klebsiella pneumoniae*; SE01: without extract; E0120: 20 mg extract; E0140: 40 mg extract and E0160: 60 mg extract.

## Data Availability

The original contributions presented in this study are included in the article. Further inquiries can be directed to the corresponding author.

## Supplementary Materials

The following supporting information can be downloaded at: https://www.mdpi.com/article/10.3390/polym18091143/s1. Figure S1: Contour plot showing the effect of xylan and PVA (AB) on elongation at Break; Figure S2: Contour plot showing the effect of xylan and glycerol (AC) on elongation at Break; Figure S3: Contour plot showing the effect of xylan and glycerol (AC) on elongation at Break; Figure S4: Contour plot showing the effect of xylan and PVA (AB) on tensile strength; Figure S5: Contour plot showing the effect of xylan and glycerol (AC) on tensile strength; Figure S6: Contour plot showing the effect of PVA and glycerol (BC) on tensile strength; Table S1: Phytochemical prospecting of the ethanolic extract of *Zanthoxylum rhoifolium*; Table S2: ANOVA for quadratic model of elongation at break; Table S3: ANOVA for quadratic model of tensile strength; Table S4: Model fit statistics; Equation (S1): Polynomial equation model for elongation at Break; Equation (S2): Polynomial equation model for tensile strength.
