# Supplementary material for: Xylan-Polyvinyl Alcohol Biopolymer Films Incorporated with Zanthoxylum rhoifolium Lam. Extract: Development, Characterization, and Antimicrobial Activity for Active Food Packaging
Source: Polymers (Basel). 2026 May 6;18(9):1143. doi: 10.3390/polym18091143 (PMC13165833; doi:10.3390/polym18091143)

Supplementary Information

Xylan-Polyvinyl alcohol Biopolymer Films Incorporated with *Zanthoxylum rhoifolium Lam*. Extract: Development, Characterization, and Antimicrobial Activity for Active Food Packaging

Janine Siqueira Nunes [1], Brunna Emanuelly Guedes de Oliveira [1], Fernanda Matias Cariri Marques [2], Abrahão Alves de Oliveira Filho [2] and Elquio Eleamen Oliveira [3*]

[1] Department of Biotechnology, Federal University of Paraíba, João Pessoa, PB, 58051-900, Brazil, janinesnunes@hotmail.com, brunnaguedes151@gmail.com.
[2] Academic Unit of Biological Sciences, Health Center and Rural Technology, Federal University of Campina Grande, Patos, PB, 58708-110, Brazil, fernandacariri20@gmail.com, abrahao.alves@professor.ufcg.edu.br
[3] Department of Biology, State University of Paraíba, Joao Pessoa, PB, 58071-470, Brazil, elquioeleamen@servidor.uepb.edu.br

Table S1. Phytochemical prospecting of the ethanolic extract of *Zanthoxylum rhoifolium.*

Metabolite class	Ethanolic extract
Alkaloids	+
Flavonoids	+
Saponins	-
Tannins	+
Terpenes/Steroids	+

Legend: (+) indicates presence (-) indicates absence of the metabolite

Table S2. Model fit statistics.

	**R^2**	**Adjusted R^2**	**Predicted R^2**
**TS**	0.9756	0.9442	0.6822
**EB**	0.9942	0.9875	0.9020

Table S3. ANOVA for quadratic model of tensile strength.

Source	Sum of Squares	df	Mean Square	F-value	p-value	
**Model**	336.30	9	37.37	31.08	< 0.0001	significant
A-xilana	29.15	1	29.15	24.24	0.0017	
B-PVA	49.45	1	49.45	41.13	0.0004	
C-glicerol	199.80	1	199.80	166.17	< 0.0001	
AB	0.1722	1	0.1722	0.1432	0.7163	
AC	41.86	1	41.86	34.81	0.0006	
BC	2.37	1	2.37	1.97	0.2030	
A^2	3.83	1	3.83	3.19	0.1175	
B^2	5.78	1	5.78	4.80	0.0645	
C^2	4.43	1	4.43	3.69	0.0963	
**Residual**	8.42	7	1.20			
Lack of Fit	6.68	3	2.23	5.12	0.0744	not significant
Pure Error	1.74	4	0.4350			
**Cor Total**	344.71	16				

Table S4. ANOVA for quadratic model of elongation at break.

Source	Sum of Squares	df	Mean Square	F-value	p-value	
**Model**	2.10E+08	8	26247.77	148.73	< 0.0001	significant
A-xilana	95992.09	1	95992.09	543.94	< 0.0001	
B-PVA	34287.94	1	34287.94	194.29	< 0.0001	
C-glicerol	25794.72	1	25794.72	146.17	< 0.0001	
AB	19136.57	1	19136.57	108.44	< 0.0001	
AC	15312.83	1	15312.83	86.77	< 0.0001	
BC	10296.39	1	10296.39	58.34	0.0001	
A^2	12280.19	1	12280.19	69.59	< 0.0001	
B^2	4951.56	1	4951.56	28.06	0.0011	
**Residual**	1235.33	7	176.48			
Lack of Fit	1176.44	3	392.15	26.63	0.0042	significant
Pure Error	58.89	4	14.72			
**Cor Total**	2.11E+08	15				

Eq. S1. Polynomial equation model for tensile strength.

$$y = 7.10778 + 0.071354 * A + 0.055329 * B - 0.184694 * C - 0.000021 * AB - 0.00107 * AC + 0.000257 * BC + 0.000095 * A^2 - 0.000117 * B^2 + 0.001140\ C^2$$

Eq. S2. Polynomial equation model for elongation at Break

$$y = 103.91844 - 0.412879 * A - 1.17425 * B + 1.83365 * C - 0.006917 * AB - 0.020624 * AC + 0.022637 * BC + 0.005877 * A^2 + 0.003800 * B^2$$

Figure S1. Contour plot showing the effect of xylan and PVA (AB) on elongation at Break.

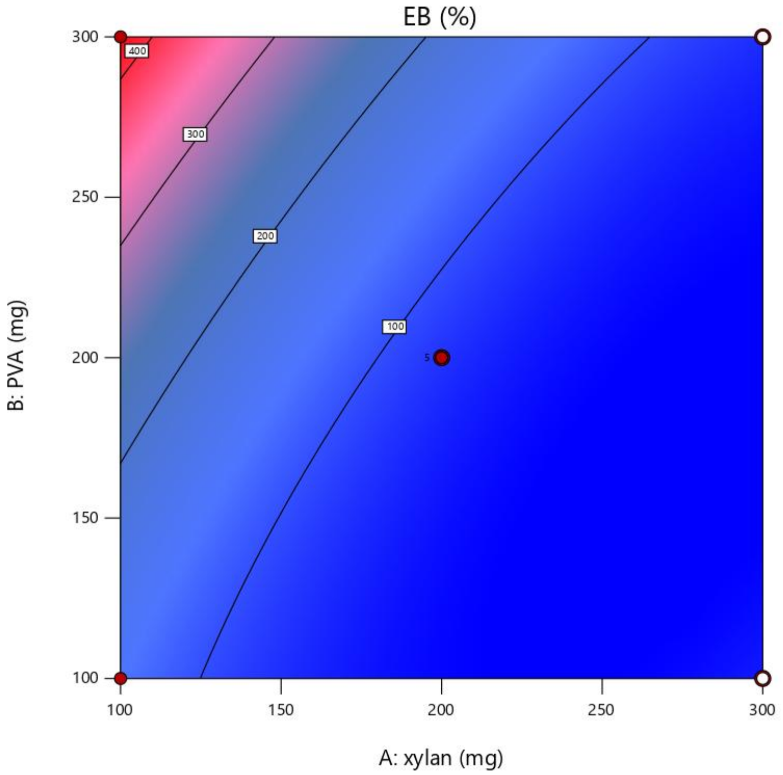

Figure S2. Contour plot showing the effect of xylan and glycerol (AC) on elongation at Break.

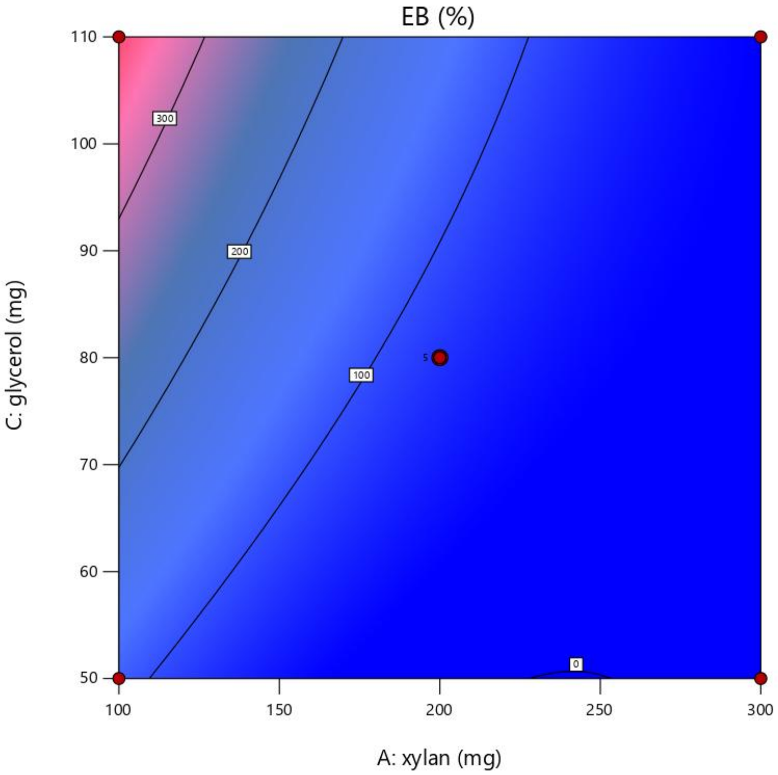

Figure S3. Contour plot showing the effect of xylan and glycerol (AC) on elongation at Break

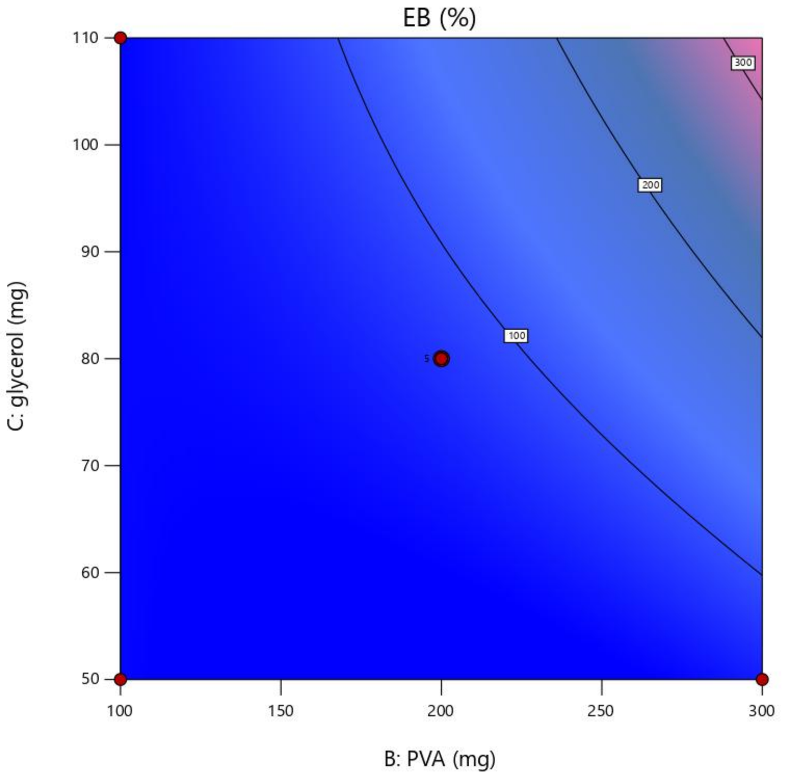

Figure S4. Contour plot showing the effect of xylan and PVA (AB) on tensile strength.

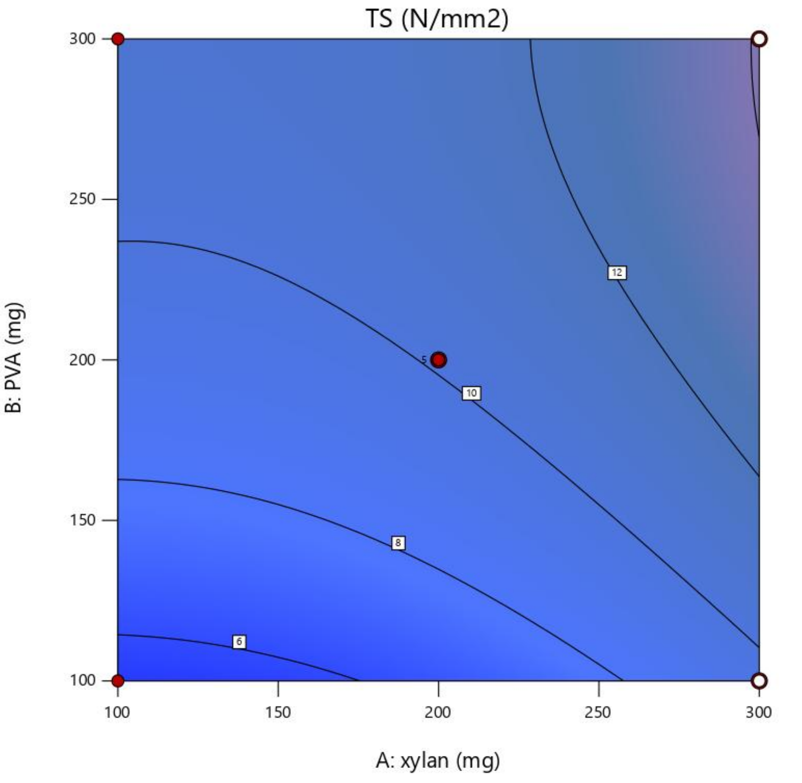

Figure S5. Contour plot showing the effect of xylan and glycerol (AC) on tensile strength.

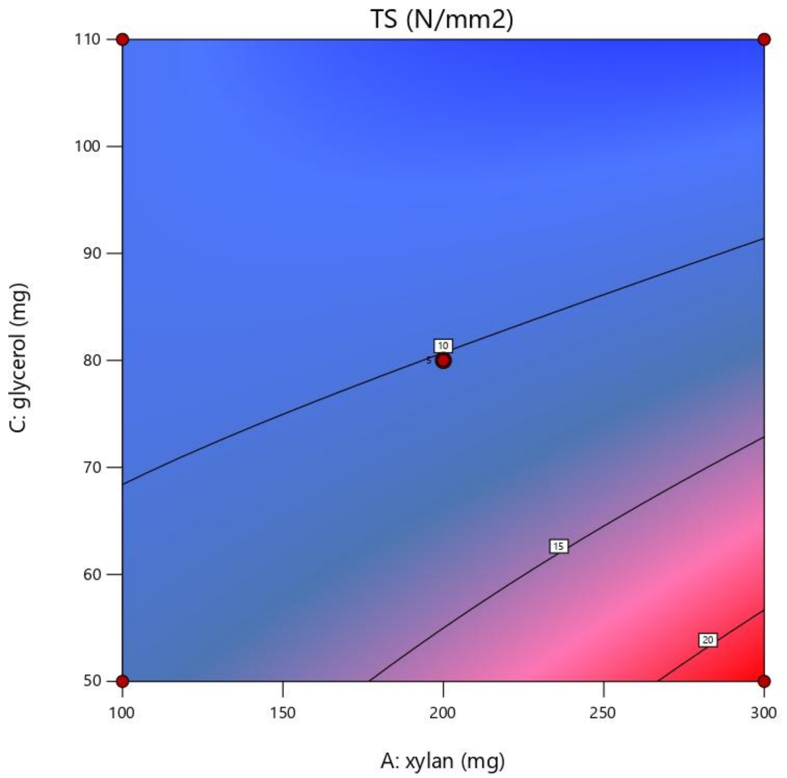

Figure S6. Contour plot showing the effect of PVA and glycerol (BC) on tensile strength.

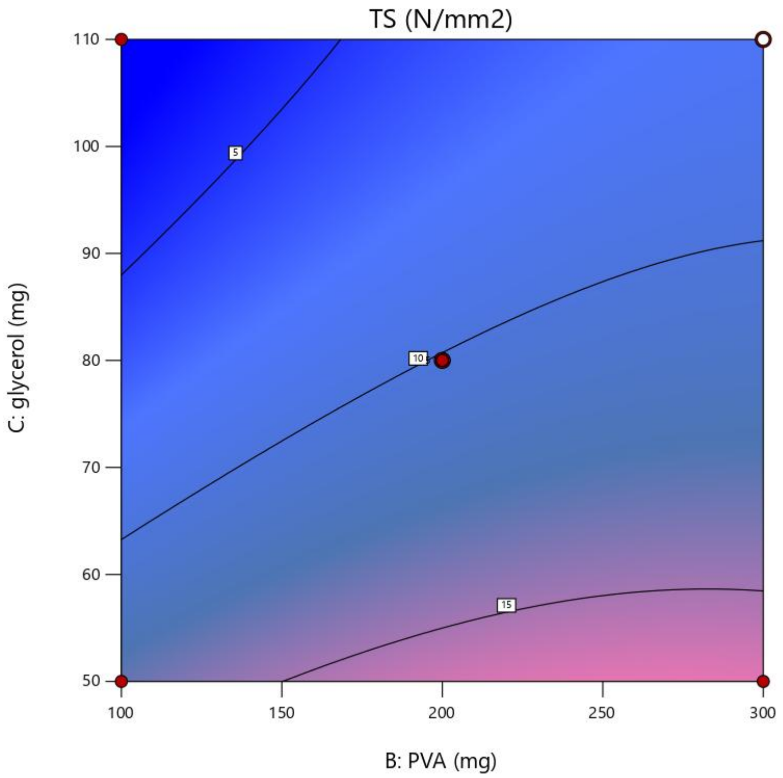

Supplement: Supplementary file 1 [file polymers-18-01143-s001.zip › polymers-4265538-supplementary.pdf]
